# Early evaluation of irradiated parotid glands with intravoxel incoherent motion MR imaging: correlation with dynamic contrast-enhanced MR imaging

**DOI:** 10.1186/s12885-016-2900-2

**Published:** 2016-11-08

**Authors:** Nan Zhou, Chen Chu, Xin Dou, Ming Li, Song Liu, Lijing Zhu, Baorui Liu, Tingting Guo, Weibo Chen, Jian He, Jing Yan, Zhengyang Zhou, Xiaofeng Yang, Tian Liu

**Affiliations:** 1 Department of Radiology, Nanjing Drum Tower Hospital, The Affiliated Hospital of Nanjing University Medical School, Nanjing, 210008 China; 2 The Comprehensive Cancer Centre of Drum Tower Hospital, Medical School of Nanjing University & Clinical Cancer Institute of Nanjing University, Nanjing, 210008 China; 3 Department of Radiology, Nanjing Drum Tower Hospital Clinical College of Traditional Chinese and Western Medicine, Nanjing University of Chinese Medicine, Nanjing, 210008 China; 4 Philips Healthcare, Shanghai, 200233 China; 5 Department of Radiation Oncology and Winship Cancer Institute, Emory University, Atlanta, GA 30322 USA

**Keywords:** Nasopharyngeal carcinoma (NPC), Parotid glands, Intravoxel incoherent motion (IVIM) MR imaging, Dynamic contrast-enhanced (DCE) MR imaging, Radiotherapy

## Abstract

**Background:**

Radiation-induced parotid damage is one of the most common complications in patients with nasopharyngeal carcinoma (NPC) undergoing radiotherapy (RT). Intravoxel incoherent motion (IVIM) magnetic resonance (MR) imaging has been reported for evaluating irradiated parotid damage. However, the changes of IVIM perfusion-related parameters in irradiated parotid glands have not been confirmed by conventional perfusion measurements obtained from dynamic contrast-enhanced (DCE) MR imaging. The purposes of this study were to monitor radiation-induced parotid damage using IVIM and DCE MR imaging and to investigate the correlations between changes of these MR parameters.

**Methods:**

Eighteen NPC patients underwent bilateral parotid T1-weighted, IVIM and DCE MR imaging pre-RT (2 weeks before RT) and post-RT (4 weeks after RT). Parotid volume; IVIM MR parameters, including apparent diffusion coefficient (ADC), pure diffusion coefficient (D), pseudo-diffusion coefficient (D*), and perfusion fraction (f); and DCE MR parameters, including maximum relative enhancement (MRE), time to peak (TTP), Wash in Rate, and the degree of xerostomia were recorded. Correlations of parotid MR parameters with mean radiation dose, atrophy rate and xerostomia degree, as well as the relationships between IVIM and DCE MR parameters, were investigated.

**Results:**

From pre-RT to post-RT, all of the IVIM and DCE MR parameters increased significantly (*p* < 0.001 for ADC, D, f, MRE, Wash in Rate; *p* = 0.024 for D*; *p* = 0.037 for TTP). Change rates of ADC, f and MRE were negatively correlated with atrophy rate significantly (all *p* < 0.05). Significant correlations were observed between the change rates of D* and MRE (*r* = 0.371, *p* = 0.026) and between the change rates of D* and TTP (*r* = 0.396, *p* = 0.017). The intra- and interobserver reproducibility of IVIM and DCE MR parameters was good to excellent (intraclass correlation coefficient, 0.633–0.983).

**Conclusions:**

Early radiation-induced changes of parotid glands could be evaluated by IVIM and DCE MR imaging. Certain IVIM and DCE MR parameters were correlated significantly.

## Background

Radiotherapy (RT) is the main treatment modality for patients with nasopharyngeal carcinoma (NPC). Radiation-induced parotid damage is one of the most common complications, causing xerostomia, dysphagia and increased risk of dental caries [1] and severely reduces the life quality of these patients. Over the past few years, intensity-modulated radiation therapy (IMRT) has been introduced for the treatment of NPC to preserve parotid function [2]. However, the parotid glands are sensitive to radiation [3], and the radiation-induced parotid damage cannot be completely avoided even with IMRT [2]. Therefore, it is important to evaluate radiation-induced parotid damage in a timely manner preferably to preserve the function of parotid glands.

The severity of xerostomia can be evaluated based on the radiation morbidity scoring criteria proposed by the Radiation Therapy Oncology Group (RTOG) [4]. However, this evaluation is subjective and cannot depict the morphological and pathophysiological changes in the irradiated parotid glands. Histological examination, which is the gold standard for the evaluation of radiation-induced parotid damage, is not suitable for routine clinical use due to its invasiveness. Scintigraphy, which reveals functional changes in irradiated parotid glands [5], involves additional radiation exposure. Magnetic resonance (MR) sialography can noninvasively depict irradiated parotid ductal damage [6], however without any parenchymal information about the irradiated parotid glands.

To investigate the structural and pathophysiological changes, dynamic contrast-enhanced (DCE) and diffusion-weighted (DW) MR imaging have been used for the evaluation of irradiated parotid glands [7–10]. Changes of the vascular permeability and extra-vascular extra-cellular space (EES) in the irradiated parotid glands can be successfully evaluated using DCE MR parameters (such as the transfer coefficient *K* ^*trans*^ and extra-vascular extra-cellular space *v*_*e*_). However, DCE MR imaging involves intravenous injection of gadolinium-based contrast agents, which cause additional expenditure and incur risks of nephrogenic systematic fibrosis or gadolinium deposition in the brain. DW MR imaging generates an apparent diffusion coefficient (ADC), which is affected by both water molecular diffusion and microvascular perfusion simultaneously.

Intravoxel incoherent motion (IVIM) MR imaging was initially proposed by Le Bihan et al. [11]. The perfusion and diffusion information can be separately extracted using IVIM MR imaging with a number of *b* values. The signal decay at low *b* values is primarily attributed to perfusion, while data obtained at high *b* values are mainly dominated by diffusion [12]. Microcirculation changes can be evaluated by IVIM perfusion-related parameters (perfusion fraction f and pseudo-diffusion coefficient D*), and the pure molecular diffusion (D) can reflect the Brownian movement of water molecules. In recent years, IVIM MR imaging has been widely used for the assessment of organic damage, differential diagnosis of tumours and monitoring of cancer therapy [13–16]. Marzi et al. found that IVIM MR parameters (ADC, ADC_low_, D and f) of parotid glands significantly changed during RT, and the changes in D values were significantly correlated with mean radiation dose [9]. This pilot study indicated the potential of IVIM MR imaging for the evaluation of radiation-induced damage to the parotid glands. However, the changes of IVIM perfusion-related parameters in this pilot study have not been confirmed by conventional perfusion measurements obtained from DCE MR imaging.

Additionally, the precise correlation between IVIM perfusion-related and DCE MR parameters has not been confirmed. Jia et al. reported a significant correlation between f and DCE MR parameters (including Enhancement Amplitude and Maximum Slope of Increase) in NPC [17]. However, Yuan et al. found no correlations between IVIM and DCE MR parameters in lung neoplasms [18]. To our knowledge, the correlations between parameters derived from IVIM and DCE MR imaging in irradiated parotid glands have never been reported.

Therefore, the purposes of this study were to observe the changes in and the relationships between IVIM and DCE MR parameters of irradiated parotid glands after RT and then to correlate the change rates in parotid IVIM and DCE MR parameters with mean radiation dose, atrophy rate and xerostomia degree.

## Methods

### Patients

**Fig. 1:**
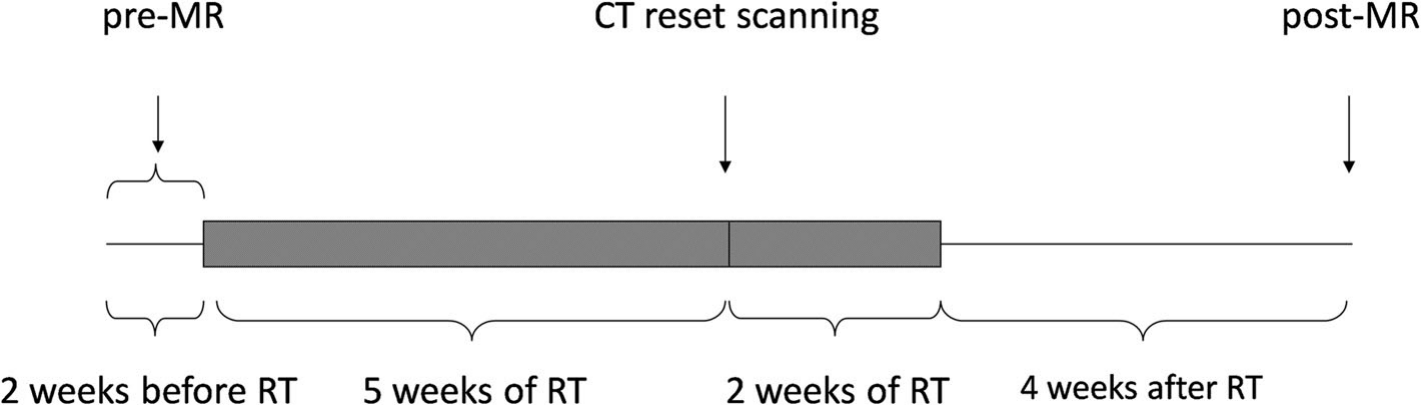
Flowchart describes the procedures of MR examinations and radiotherapy (RT) for patients with nasopharyngeal carcinoma. Pre-MR and post-MR are the MR examinations within 2 weeks before RT (pre-RT) and 4 weeks after RT (post-RT), respectively. CT reset scanning proceeded approximately 5 weeks after the beginning of RT for the formulation of the second course RT scheme

This study was approved by the institutional review board of our hospital, and all of the patients provided written informed consent. From August 2015 to April 2016, 18 patients (male, 14; female, 4; age, 24–70 years old; mean age, 50.1 ± 10.3 years) with an initial diagnosis of poorly differentiated NPC were prospectively enrolled. The inclusion criteria were: (1) a pathological diagnosis of poorly differentiated NPC through biopsy and readiness to receive IMRT with concurrent chemotherapy in our hospital; (2) scheduled to undergo MR evaluation and follow-up in our hospital; and (3) no any history of allergy to gadolinium contrast agents, with a glomerular filtration rate greater than 30 mL/min to accommodate the injection of contrast agents. The exclusion criteria included: (1) absolute MR examination contraindications, such as cardiac pacemakers, aneurysm clips, artificial cochlea implantation, etc.; (2) a history of parotid disorders, such as parotitis, parotid tumours, etc.; and (3) having received RT to the head and neck region in the past. The patients’ characteristics and the flowchart of this study are shown in Table 1 and Fig. 1, respectively.

**Table 1:** Clinical data of NPC patients undergoing radiotherapy

NO.	Age ranges (years)	Radiation dose (Gy)		Xerostomia degree	
		R	L	Pre-RT	Post-RT
1	51–60	28.5	27.5	0	1
2	61–70	25.6	26.9	0	2
3	41–50	26.0	25.9	0	1
4	41–50	25.8	25.0	0	2
5	51–60	25.5	25.9	0	1
6	21–30	32.6	34.3	0	1
7	51–60	30.4	33.2	0	2
8	61–70	30.3	29.9	0	1
9	61–70	27.7	28.8	0	1
10	41–50	28.1	28.1	0	1
11	51–60	31.0	29.7	0	2
12	51–60	30.8	31.5	0	2
13	41–50	29.0	28.8	0	2
14	41–50	27.6	27.0	0	1
15	51–60	29.9	30.9	0	2
16	41–50	25.3	24.8	0	2
17	41–50	25.8	26.7	0	2
18	41–50	28.9	27.9	0	1

*NPC* nasopharyngeal carcinoma, *pre*-*RT* approximately 2 weeks before radiotherapy (*RT*), *post*-*RT* approximately 4 weeks after RT, *R* right parotid gland, *L* left parotid gland. Xerostomia degree was evaluated by the acute radiation morbidity scoring criteria proposed by the Radiation Therapy Oncology Group (*RTOG*)

All of the patients were treated with IMRT to the nasopharyngeal lesions and the neck lymphatic drainage areas, combined with concurrent chemotherapy (three cycles; nedaplatin 60 mg for each cycle). The total accumulated radiation dose within the tumour region was 70Gy, which was divided into two courses. In the first course, the RT field covered the nasopharyngeal lesions and neck lymphatic drainage areas (25 fractions; 2 Gy for each fraction). In the second course, the RT field was reduced to the tumour area according to the CT reset condition (10 fractions; 2 Gy for each fraction). The RT was administered as one fraction for 1 day, five fractions for 1 week and a total of 35 fractions for 7 weeks. Because the bilateral parotid and submandibular glands were quite close to the planning target volume (PTV), some attempt was undertaken to reduce their radiation doses on the premise of meeting the tumour exposure dose. The mean accumulated radiation dose to the parotid glands was calculated from the treatment planning system of Pinnacle^3^ (Philips Medical Systems, Fitchburg, WI, USA) and TomoTherapy HiArt (TomoTherapy, Madison, WI, USA). Because the bilateral parotid glands received different radiation doses during the course of RT, the bilateral parotid glands of each patient were analysed separately. The mean total accumulated radiation dose to the parotid glands was 28.4 ± 2.4 Gy after RT, which was less than our hospital limit for parotid radiation dose of 30–35 Gy for 50 % volume. All of the patients underwent two MR examinations within 2 weeks before RT (pre-RT) and 4 weeks after RT (post-RT), and the MR scan protocol remained identical during the course. All of the patients successfully underwent the whole therapy and follow-up MR examinations.

### Clinical assessment of xerostomia

The degree of xerostomia in NPC patients was assessed 1 hour prior to each MR examination by a radiation oncologist (X.X., with 10 years of clinical experience in head and neck RT), according to the acute radiation morbidity scoring criteria proposed by the Radiation Therapy Oncology Group (RTOG) [4]: grade 0 is no change over baseline; grade 1 indicates mild mouth dryness or slightly thickened saliva but without alterations in the baseline feeding behaviour, such as the increased use of liquids with meals; grade 2 represents moderate dryness or sticky saliva; grade 3 indicates complete dryness; and grade 4 characterizes acute salivary gland necrosis. The grade of xerostomia in each patient at each time was recorded.

### MR imaging

All of the patients were asked to fast for 2 h before each MR examination. A full digital 3.0 T MR scanner (Ingenia, Philips Medical Systems, Best, the Netherlands) was used for the MR examinations, with a 16-channel head/neck phased array coil. Patients were placed in the supine position with the head first. The scanning sequences included: transverse T1-weighted (T1W) imaging, intravoxel incoherent motion (IVIM) MR imaging and dynamic contrast-enhanced (DCE) MR imaging. The total duration of the MR examination was approximately 10 min, 9 s.

T1W imaging was obtained with a turbo spin-echo (TSE) sequence. The other parameters were as follows: repetition time/echo time: 400–675 msec/18 msec; TSE factor: 8; matrix: 276 × 215; slice thickness: 5 mm; slice gap: default; slices: 38; field of view: 22 cm; voxel size: 0.8 mm × 0.92 mm; and number of signals averaged: 2. The duration of T1W imaging was approximately 2 min, 27 s.

IVIM MR imaging was obtained with a single-shot echo-planar imaging (SS-EPI) sequence with spectral presaturation attenuated inversion recovery (SPAIR) fat suppression before the injection of the gadolinium contrast agent. A volume shim covering the region of bilateral parotid glands was used to minimize susceptibility artefacts. The other parameters were as follows: repetition time/echo time: 6000 msec/shortest; matrix: 84 × 104; slice thickness: 4 mm; slice gap: 0.4 mm; slices: 22; field of view:22 cm; voxel size: 2.5 mm × 2.08 mm; and number of signals averaged: 2. A total of 9 *b* values (0, 25, 50, 75, 100, 150, 200, 500, and 800 s/mm^2^) were applied in the IVIM MR imaging. The duration of the IVIM MR imaging sequence was approximately 5 min, 6 s.

DCE MR imaging was obtained with a three-dimensional (3D) T1-fast field echo (FFE) sequence. Intravenous bolus injection of gadodiamide (0.2 mL/kg bodyweight, GE Healthcare Ireland, Shanghai, China) was administered at a rate of 3.0 mL/s followed by a 15 mL saline flush using an automatic power injection (Medrad Spectris Solaris EP MR Injector System; One Medrad Drive Indianola, PA, USA). The other parameters were as follows: repetition time/echo time: shortest/shortest; flip angle: 10°; matrix: 232 × 232; slice thickness: 2 mm; slice gap: default; slices: 68; field of view:30 cm; voxel size: 1.3 mm × 1.29 mm; and number of signals averaged: 1. A total of 15 dynamics at an interval of 10.4 s were obtained for each patient. The duration of the DCE MR sequence was approximately 2 min, 36 s.

All of the patients completed all of the MR examinations successfully without any discomfort or side effects.

### Image analysis

All the MR images were analysed and measured independently by 2 radiologists (X.X., X.X.X.) with 3 and 11 years of experience, respectively, in head and neck radiology, who were blinded to the clinical information of all of the patients. Averaged values of the two radiologists’ measurements were treated as the final results for the parotid glands.

T1W images were transferred into a workstation (Extended MR WorkSpace 2.6.3.5, Philips Medical Systems, Best, the Netherlands), which was used to calculate the parotid volume owing to its perfect soft tissue contrast. The outline of each parotid gland was drawn slice by slice on T1W images to obtain the area of each slice. The volume of each parotid gland was calculated by the following equation: V = ∑S_*i*_ × (ST + SG), where V represents the volume of the parotid gland, S_*i*_ represents the area of the *i*th slice in the parotid gland, ST represents the slice thickness, and SG represents the slice gap. The atrophy rate of each parotid gland from pre-RT to post-RT was calculated by the following equation: R_V_ = (V_pre_–V_post_) / V_pre_ × 100 %, where R_V_ is the atrophy rate of the parotid gland, and V_pre_ and V_post_ are the parotid volume pre-RT and post-RT, respectively.

IVIM MR images were post-processed using DWI-Tool, developed by Philips in IDL 6.3 (ITT Visual Information Solutions, Boulder, CO, USA). D, D* and f maps were generated using the bi-exponential fit equation [19]: S_*b*_/S_0_ = (1–f) ∙ exp (−*b*D) + f ∙ exp [−*b* (D + D*)], where S_*b*_ represents the mean signal intensity at different *b* values of 25, 50, 75, 100, 150, 200, 500, and 800 s/mm^2^, S_0_ represents the mean signal intensity at a *b* value of 0 s/mm^2^, f represents the fraction of diffusion linked to microcirculation, exp is exponential, D represents the slow component of diffusion, and D* represents the fast component of diffusion. The ADC map was generated using the mono-exponential fit equation [19]: ln (S_*b*_) = ln (S_0_)−*b*ADC, where ADC represents the microscopic translational motions, including the pure molecular diffusion and perfusion-related diffusion. A region of interest (ROI) was delineated manually on the largest slice of the IVIM MR images for each parotid gland to include as much as parotid parenchyma without obvious vessels. The ROI was automatically transferred between mono-exponential and bi-exponential models, and the corresponding D, D*, f and ADC values of the parotid glands were obtained. The change rates of D, D*, f and ADC values were calculated by the following equation: R_IVIM-PARs_ = (IVIM-PAR_post_−IVIM-PAR_pre_) / IVIM-PAR_pre_ × 100 %, where R_IVIM-PARs_ is the change rate of D, D*, f and ADC values from pre-RT to post-RT, and IVIM-PAR_post_ and IVIM-PAR_pre_ are the D, D*, f and ADC values post-RT and pre-RT, respectively.

DCE MR images were post-processed using the “Basic T1 Perfusion” function on the aforementioned workstation. The time-intensity curve was depicted automatically, and the DCE MR parameters, including the maximum relative enhancement (MRE), time to peak (TTP) and Wash in Rate, were calculated. MRE is the maximal signal enhancement of a pixel of certain dynamic relative to that same pixel with the pre-contrast dynamic. TTP is the time between the time of initial intensity and the time of peak intensity. Wash in Rate is the maximum slope between the time of initial intensity and the time of peak intensity. An ROI was drawn manually on the largest slice of the parotid gland to include as much as parotid parenchyma with visible vessels excluded, and the corresponding MRE, TTP and Wash in Rate were automatically obtained. The change rates of the MRE, TTP and Wash in Rate were calculated by the following equation: R_DCE-PARs_ = (DCE-PAR_post_−DCE-PAR_pre_) / DCE-PAR_pre_ × 100 %, where R_DCE-PARs_ is the change rate of MRE, TTP and Wash in Rate from pre-RT to post-RT, and DCE-PAR_post_ and DCE-PAR_pre_ are the MRE, TTP and Wash in Rate post-RT and pre-RT, respectively.

The IVIM and DCE MR parameters were repeatedly measured by the second observer with an interval of 4 weeks between measurements to evaluate intraobserver reproducibility.

### Statistical analysis

Continuous numeric data with normal distribution are reported as the means ± standard deviations (SD). The paired sample *t* test was used to identify any significant changes of IVIM and DCE MR parameters from pre-RT to post-RT. The differences of averaged bilateral parotid R_IVIM-PARs_ and R_DCE-PARs_ between grade 1 and grade 2 of the post-RT xerostomia degree were analysed using the independent-samples *t* test. Pearson’s correlation test was used to detect the correlations between the change rates of parotid IVIM and DCE MR parameters and mean radiation dose or atrophy rates, as well as between the change rates of IVIM and DCE MR parameters. The intra- and interobserver reproducibility of IVIM and DCE MR parameters was evaluated by calculating intraclass correlation coefficient (ICC) values. The ICC was between 0 and 1, and the interpretation of ICC was as follows: < 0.20, poor; 0.21–0.40, fair; 0.41–0.60, moderate; 0.61–0.80, good; and > 0.80, excellent [20]. Statistical analysis was performed using SPSS software, version 16.0 (SPSS Inc., Chicago, IL, USA). A two-tailed *p* values < 0.05 was considered statistically significant.

## Results

**Fig. 2:**
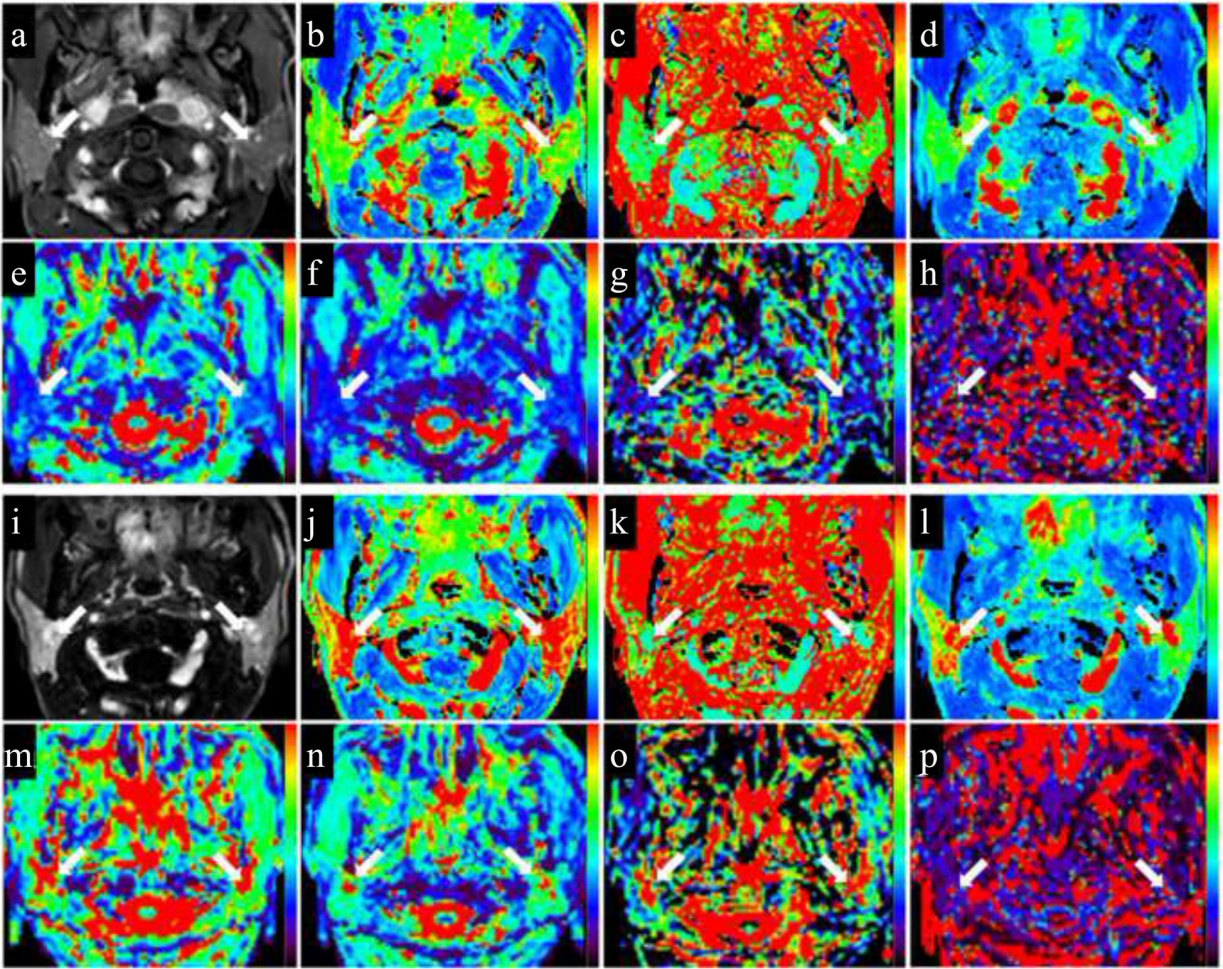
MR images of bilateral parotid glands (*arrows*) in one patient with nasopharyngeal carcinoma (NPC). **a**-**h** Dynamic contrast-enhanced (DCE, **a**-**d**) and intravoxel incoherent motion (IVIM, **e**-**h**) MR images within 2 weeks before radiotherapy (pre-RT). **i**-**p** DCE (**i**-**l**) and IVIM (**m**-**p**) MR images approximately 4 weeks after radiotherapy (post-RT). At pre-RT, the right and left parotid maximum relative enhancement (MRE, **b**), time to peak (TTP, **c**), Wash in Rate (**d**), apparent diffusion coefficient (ADC, **e**), pure diffusion coefficient (D, **f**), perfusion fraction (**f**, **g**), and pseudo-diffusion coefficient (D*, **h**) values are 222.8 and 243.7 %, 46.8 s and 52.0 s, 143.9 i/s and 100.7 i/s, 0.76 × 10^−3^ mm^2^/s and 0.85 × 10^−3^ mm^2^/s, 0.69 × 10^−3^ mm^2^/s and 0.73 × 10^−3^ mm^2^/s, 0.089 and 0.116, and 50.8 × 10^−3^ mm^2^/s and 32.2 × 10^−3^ mm^2^/s, respectively. At post-RT, the right and left parotid MRE (**j**), TTP (**k**), Wash in Rate (**l**), ADC (**m**), D (**n**), f (**o**), and D* (**p**) values are 335.6 and 357.9 %, 62.6 s and 83.5 s, 237.0 i/s and 146.4 i/s, 1.70 × 10^−3^ mm^2^/s and 1.59 × 10^−3^ mm^2^/s, 1.41 × 10^−3^ mm^2^/s and 1.31 × 10^−3^ mm^2^/s, 0.184 and 0.175, and 54.3 × 10^−3^ mm^2^/s and 39.0 × 10^−3^ mm^2^/s, respectively

From pre-RT to post-RT, the volume of bilateral parotid glands significantly decreased from 26.1 ± 5.5 cm^3^ to 18.9 ± 3.9 cm^3^, with an atrophy rate of 26.5 ± 10.3 % (*p* < 0.001). The pre- and post-RT IVIM and DCE MR images of the bilateral parotid glands in one representative NPC patient are shown in Fig. 2.

### Changes of IVIM and DCE MR parameters from pre-RT to post-RT

As shown in Table 2, all of the IVIM and DCE MR parameters increased from pre-RT to post-RT significantly (all *p* < 0.05).

**Table 2:** The IVIM and DCE MR parameters of parotid glands pre-RT and post-RT

	pre-RT	post-RT	*p* value
IVIM MR parameters			
ADC (10^−3^ mm^2^/s)	0.88 ± 0.15	1.45 ± 0.20	<0.001^a^
D (10^−3^ mm^2^/s)	0.72 ± 0.13	1.16 ± 0.21	<0.001^a^
D* (10^−3^ mm^2^/s)	36.3 ± 22.7	50.8 ± 27.4	0.024^a^
f (%)	13.3 ± 3.9	19.2 ± 5.6	<0.001^a^
DCE parameters			
MRE (%)	182.0 ± 41.3	287.1 ± 41.4	<0.001^a^
TTP (s)	42.4 ± 28.9	53.7 ± 26.0	0.037^a^
Wash in Rate (i/s)	97.5 ± 44.4	159.8 ± 54.5	<0.001^a^

*IVIM* Intravoxel incoherent motion, *DCE* Dynamic contrast-enhanced, *pre*-*RT* Approximately 2 weeks before radiotherapy (RT), *post*-*RT* approximately 4 weeks after RT, *ADC* Apparent diffusion coefficient, *D* Pure diffusion coefficient, D* Pseudo-diffusion coefficient, *f* perfusion fraction, *MRE* Maximum relative enhancement, *TTP* Time to peak

^a^denotes a significant difference between pre-RT and post-RT parameters

### Correlations between changes of IVIM or DCE MR parameters and atrophy rate (and mean radiation dose)

**Fig. 3:**
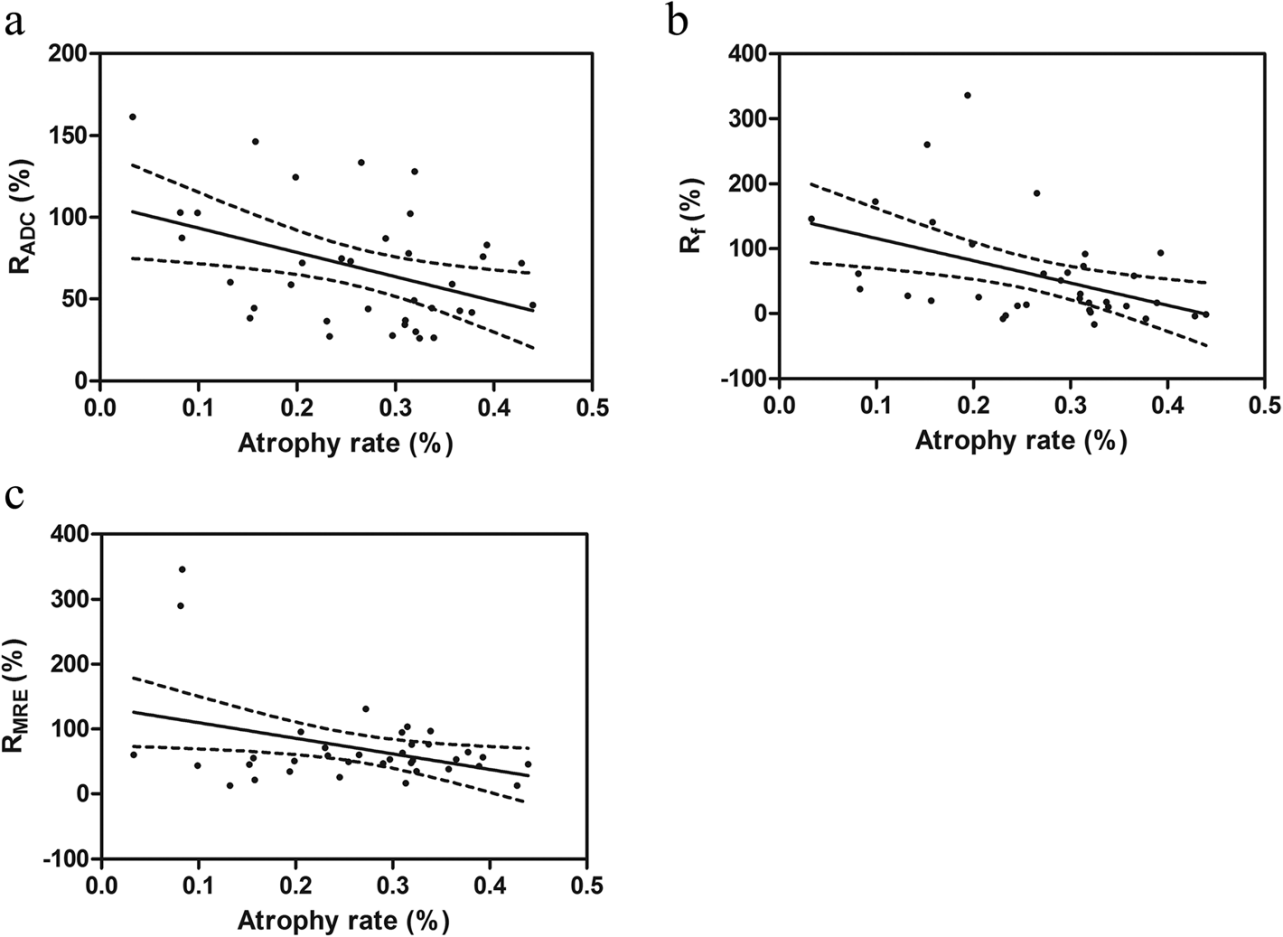
Scatter plots show that the change rates of apparent diffusion coefficient (ADC) (**a**), perfusion fraction (f) (**b**) and maximum relative enhancement (MRE) (**c**) values are significantly correlated with the parotid atrophy rate from pre-RT (approximately 2 weeks before radiotherapy) to post-RT (approximately 4 weeks after radiotherapy). R_ADC_, R_f_ and R_MRE_ are the change rates of ADC, f and MRE values from pre-RT to post-RT, respectively. The dashed lines are the 95 % confidence bands

As shown in Table 3 and Fig. 3, the change rates of parotid ADC, f and MRE were negatively correlated with the atrophy rate significantly from pre-RT to post-RT (all *p* < 0.05). There was no significant correlation between mean radiation dose and any change rate of parotid IVIM or any DCE MR parameter (all *p* > 0.05).

**Table 3:** Correlations between atrophy rate and the change rates of parotid IVIM or DCE MR parameters

Atrophy rate	r	*p* value
R_IVIM-PARs_		
R_ADC_	−0.418	0.011^a^
R_D_	0.066	0.702
R_D*_	0.038	0.825
R_f_	−0.449	0.006^a^
R_DCE-PARs_		
R_MRE_	−0.372	0.025^a^
R_TTP_	0.208	0.223
R_Wash in Rate_	−0.136	0.428

*IVIM* Intravoxel incoherent motion, *DCE* Dynamic contrast-enhanced, *r* Pearson correlation coefficient, *R*_*IVIM*-*PARs*_ *and R*_*DCE*-*PARs*_ are the change rates of IVIM and DCE MR parameters from pre-RT to post-RT, respectively, *R*_*ADC*_, *R*_*D*_, *R*_D*_, *R*_*f*_, *R*_*MRE*_, *R*_*TTP*_ *and R*_*Wash in Rate*_ are the change rates of apparent diffusion coefficient (ADC), pure diffusion coefficient (D), pseudo-diffusion coefficient (D*), perfusion fraction (f), maximum relative enhancement (MRE), time to peak (TTP) and Wash in Rate from pre-RT (2 weeks before radiotherapy) to post-RT (4 weeks after RT), respectively

^a^denotes a significant correlation between the parotid atrophy rate and the change rate of each IVIM or DCE MR parameter

### Relationships between IVIM or DCE MR parameters and xerostomia degree

The average change rates of bilateral parotid IVIM and DCE MR parameters in patients with grade 1 xerostomia degree did not significantly differ from that in patients with grade 2 (all *p* > 0.05).

### Correlations between IVIM and DCE MR parameters

As shown in Table 4, the change rate of D* was correlated with that of MRE (*r* = 0.371 and *p* = 0.026) and TTP (*r* = 0.396 and *p* = 0.017) significantly from pre-RT to post-RT, and there were no significant correlations between the change rates of other IVIM and DCE MR parameters.

**Table 4:** Correlations between change rates of parotid IVIM and DCE MR parameters

R_IVIM-PARs_	R_DCE-PARs_	*r*	*p* value
R_ADC_	R_MRE_	0.103	0.550
	R_TTP_	−0.048	0.780
	R_Wash in Rate_	−0.006	0.973
R_D_	R_MRE_	0.191	0.264
	R_TTP_	0.156	0.364
	R_Wash in Rate_	0.181	0.290
R_D*_	R_MRE_	0.371	0.026^a^
	R_TTP_	0.396	0.017^a^
	R_Wash in Rate_	0.251	0.139
R_f_	R_MRE_	−0.079	0.647
	R_TTP_	−0.238	0.162
	R_Wash in Rate_	−0.116	0.500

*IVIM* Intravoxel incoherent motion, *DCE* dynamic contrast-enhanced, *r* Pearson correlation coefficient, *R*_*IVIM*-*PARs*_ *and R*_*DCE*-*PARs*_ are the change rates of IVIM and DCE MR parameters from pre-RT to post-RT, respectively, *R*_*ADC*_, *R*_*D*_, *R*_D*_, *R*_*f*_, *R*_*MRE*_, *R*_*TTP*_ *and R*_*Wash in Rate*_ are the change rates of apparent diffusion coefficient (ADC), pure diffusion coefficient (D), pseudo-diffusion coefficient (D*), perfusion fraction (f), maximum relative enhancement (MRE), time to peak (TTP) and Wash in Rate from pre-RT (2 weeks before radiotherapy) to post-RT (4 weeks after RT), respectively

^a^denotes a significant correlation between the change rates of IVIM and DCE MR parameters

### Reproducibility of IVIM and DCE MR parameters

As shown in Table 5, the measurements of most parotid IVIM and DCE MR parameters showed excellent intra- and interobserver agreement (ICC, 0.911–0.983), except it was good for f and D* (ICC, 0.633–0.793).

**Table 5:** Intra- and interobserver agreement (ICC) for the measurements of parotid IVIM and DCE MR parameters

	Intraobserver ICC	Interobserver ICC
IVIM MR parameters		
ADC	0.935 (0.895–0.959)	0.930 (0.889–0.956)
D	0.939 (0.902–0.962)	0.935 (0.896–0.959)
D*	0.793 (0.608–0.890)	0.638 (0.421–0.773)
f	0.655 (0.449–0.784)	0.633 (0.413–0.770)
DCE MR parameters		
MRE	0.978 (0.965–0.986)	0.966 (0.933–0.983)
TTP	0.914 (0.863–0.964)	0.911 (0.826–0.955)
Wash in Rate	0.983 (0.974–0.990)	0.980 (0.960–0.990)

*IVIM* Intravoxel incoherent motion, *DCE* Dynamic contrast-enhanced, *ICC* Intraclass correlation coefficient, *ADC* Apparent diffusion coefficient, *D* pure diffusion coefficient, D* pseudo-diffusion coefficient, *f* perfusion fraction, *MRE* maximum relative enhancement, *TTP* Time to peak. The numbers between parentheses are 95 % confidence intervals

## Discussion

Xerostomia, which is caused by irradiated parotid damage, is a common complication in NPC patients receiving RT. Morphological and microstructural changes in irradiated parotid glands can be noninvasively evaluated by MR imaging [6, 7, 10]. Tissue perfusion (D*, f) and water molecular diffusion (D) features can be quantitatively characterized by IVIM MR imaging with bi-exponential algorithms [11], and tissue perfusion information about the microcirculation can be described with semiquantitative DCE MR imaging [21, 22]. In this study, the changes in irradiated parotid glands from pre-RT to post-RT were successfully monitored by IVIM and DCE MR parameters, and correlations between the change rates of IVIM and DCE MR parameters were confirmed.

All of the averaged bilateral parotid IVIM MR parameters (including ADC, D, D* and f) increased significantly from pre-RT to post-RT in this study. Marzi et al. reported significant increases of parotid ADC, D, ADC_low_, and f values in patients with head and neck cancer from baseline to the completion of RT [9], consistent with our results. The significant increase of ADC and D values might result from the widespread necrosis of acinar cells induced by RT [23], which caused lower cell density and an augmentation of water molecular diffusion. Houweling et al. reported a significant increase in *v*_*e*_ due to cell loss at 6 weeks after RT in oropharyngeal cancer patients [7], in accordance to our hypothesis. Marzi et al. attributed the increases in parotid ADC_low_ and f on the same day of the completion of RT to radiation-induced vascular oedema, which caused vasodilation and an increase in blood volume [9]. We speculated that the increases of D* and f values in our study shared the same pathophysiologic mechanism. Although Xu et al. reported that parotid microvascular density decreased at 4 h after RT [24], we considered the increase of blood volume secondary to vascular oedema to be the main effect of RT in the early phase of radiation-induced parotid damage. Furthermore, Lee et al. documented a significant increase in parotid vascular plasma volume (*v*_*p*_) at 3 months after RT in patients with head and neck cancer, explained by vasodilatation and increased blood volume induced by inflammation [25]; we share the same opinion as them.

All of the averaged bilateral parotid DCE MR parameters (including MRE, TTP and Wash in Rate) increased significantly from pre-RT to post-RT in this study. The increase of MRE and Wash in Rate might share the same mechanism that caused the increase of D* and f values, that is, vascular oedema and increased blood volume secondary to inflammation after RT. In addition, the augmentation of EES due to cell loss also promoted the accumulation of contrast agent in parotid tissue. Juan et al. found a significantly higher TTP value in irradiated parotid glands compared with non-irradiated glands [8], which was in agreement with our observation.

From pre-RT to post-RT, significant correlations were found between parotid atrophy rate and the change rates of ADC, f and MRE, while Marzi et al. also reported a significant correlation between the change rate of ADC values and parotid atrophy rate [9]. However, the correlations were negative in our study and were positive in Marzi et al.’s, probably due to the discrepancy in follow-up time points between the two studies. The follow-up time point in our study was 4 weeks after RT, while Marzi et al. chose the same day as the completion of RT. Four weeks after RT, interstitial fibrosis was observed in irradiated parotid glands [26], which might have contributed to the negative correlation between the change rate of ADC value and the parotid atrophy rate at 4 weeks after RT. Interstitial fibrosis could not fully compensate for the increase in EES secondary to radiation-induced parotid cell loss, but it reduced the augmentation of the ADC value induced by the increased EES. Moreover, greater parotid atrophy, indicating more severe damage, was usually accompanied by more interstitial fibrosis. Therefore, a greater parotid atrophy rate might induce a smaller change rate in ADC value due to the increase in interstitial fibrosis, although Marzi et al. also reported a significant, positive correlation between the change rate of f and the parotid atrophy rate on the same day as the completion of RT [9], in contradiction of our observations as well. This difference might also have resulted from the different MR examination time points between us. Xu et al. reported that the parotid microvascular density in miniature pigs decreased by approximately 20 and 40 % at 4 h and 2 weeks after irradiation (25 Gy), respectively [24]. According to this finding, we could speculate that a greater parotid atrophy rate might be accompanied by more severe microvascular damage at 4 weeks after RT. In our opinion, radiation-induced vascular oedema was the main effect in the early phase after RT, and the decreased microvascular density could not adequately compensate for and merely reduced the increase in blood volume induced by vascular oedema. Therefore, a greater parotid atrophy rate was accompanied by smaller change rates of f and MRE from pre-RT to post-RT.

Good to excellent intra- and interobserver agreement of parotid IVIM and DCE parameters was confirmed in this study. Excellent intra- and interobserver agreement of parotid IVIM MR parameters (D, D*, f) was reported by Su et al. and Xu et al. in healthy volunteers and in patients with Sjögren’s Syndrome, respectively [20, 27]. However, Patel et al. reported good to excellent reproducibility for ADC, D and f and fair reproducibility for D* on liver IVIM MR imaging [28]. The poor reproducibility of D* might have been due to the underlying breathing motion artefacts on liver MR imaging, which were absent on parotid MR imaging. In contrast, IVIM perfusion-related parameters are mainly affected by low *b* values (*b* ≤ 100 s/mm^2^) [12]. Five low *b* values (0, 25, 50, 75, 100 s/mm^2^) were adopted in our study, while Patel et al. used only three low *b* values (0, 50, 100 s/mm^2^) for liver IVIM MR imaging, which might be another reason for the poor reproducibility of D* measurement in their study.

Based on our findings in this study, the radiation-induced changes in the parotid microstructure could be reflected by IVIM diffusion-related parameters (ADC, D) noninvasively and quantitatively, and the radiation-induced microvascular changes could be assessed by IVIM perfusion-related parameters (f, D*), while parotid IVIM perfusion-related parameters shared similar change patterns with DCE MR parameters from pre-RT to post-RT, which indicated that IVIM perfusion-related parameters could serve as an alternative to DCE MR parameters in the evaluation of irradiated parotid microvascular damage, especially for those patients with renal insufficiency. Both IVIM and DCE MR imaging could serve as objective modalities for evaluating irradiated parotid damage, rather than subjective evaluation of the degree of xerostomia. The change rates of parotid ADC, f and MRE values were negatively correlated with the atrophy rate of the parotid gland from pre-RT to post-RT. It was reported that the decreased parotid gland volume was significantly correlated with decreased saliva production in patients with head-and-neck cancer undergoing RT [29]. The correlations between IVIM or DCE MR parameters and parotid function indices (such as saliva production) will be investigated in our future study, and long-term follow-up will be performed to explore the prognostic values of IVIM and DCE MR parameters in evaluating the damage of irradiated parotid glands. Based on the findings of IVIM or DCE MR imaging, radiation oncologists could perform early interventions to avoid long-term irreversible damage to the parotid glands in NPC patients undergoing RT.

Our study had several limitations. Firstly, the sample size was relatively small although larger than in some other studies of MR imaging in the evaluation of radiation-induced parotid damage [6, 10], and a larger cohort of patients for the correlation of the change rates of IVIM and DCE MR parameters might have been more reliable. Secondly, the range of the mean parotid radiation dose was relatively limited, which might have obscured the relationships between the mean radiation dose and parotid IVIM or DCE MR parameters. We aim to enrol more patients with other head and neck tumours to enlarge the range of the mean parotid radiation dose in the future. Thirdly, the pathological information of irradiated parotid glands in NPC patients was absent in this study due to its invasiveness. Hence, we should perform animal experiments to confirm our hypotheses.

## Conclusions

IVIM and DCE MR imaging can noninvasively evaluate the pathophysiologic changes in irradiated parotid glands, including necrosis of acinar cells, vascular oedema and interstitial fibrosis. The IVIM and DCE MR parameters in irradiated parotid glands shared the same change patterns, and significant correlations were found between the change rates of D* and MRE or TTP. IVIM and DCE MR imaging could serve as objective, quantitative and noninvasive modalities for evaluating irradiated parotid damage.

## Abbreviations

ADC: Apparent diffusion coefficient
D*: Pseudo-diffusion coefficient
D: Pure molecular diffusion
DCE: Dynamic contrast-enhanced
DW: Diffusion-weighted
EES: Extra-vascular extra-cellular space
F: Perfusion fraction
ICC: Intraclass correlation coefficient
IMRT: Intensity-modulated radiation therapy
IVIM: Intravoxel incoherent motion
MR: Magnetic resonance
MRE: Maximum relative enhancement
NPC: Nasopharyngeal carcinoma
post-RT: 4 weeks after radiotherapy
pre-RT: 2 weeks before radiotherapy
R_DCE-PARs_: Change rates of MRE, TTP and Wash in Rate from pre-RT to post-RT
R_IVIM-PARs_: Change rates of D, D*, f and ADC values from pre-RT to post-RT
ROI: Region of interest
RT: Radiotherapy
T1W: T1-weighted
TTP: Time to peak
